# Bridging the Gap: Integrating 3D Bioprinting and Microfluidics for Advanced Multi-Organ Models in Biomedical Research

**DOI:** 10.3390/bioengineering11070664

**Published:** 2024-06-28

**Authors:** Marco De Spirito, Valentina Palmieri, Giordano Perini, Massimiliano Papi

**Affiliations:** 1 Department of Neuroscience, Universita Cattolica del Sacro Cuore, Largo Francesco Vito 1, 00168 Rome, Italy; marco.despirito@unicatt.it (M.D.S.); valentina.palmieri@cnr.it (V.P.); giordano.perini@unicatt.it (G.P.); 2 Istituti di Ricovero e Cura a Carattere Scientifico IRCSS, Fondazione Policlinico Universitario “A. Gemelli”, Largo A. Gemelli 8, 00168 Rome, Italy; 3 Istituto dei Sistemi Complessi, Consiglio Nazionale delle Ricerche, CNR, via dei Taurini 19, 00185 Rome, Italy

**Keywords:** 3D printing, microfluidics, multi-organ, lab-on-chip

## Abstract

Recent advancements in 3D bioprinting and microfluidic lab-on-chip systems offer promising solutions to the limitations of traditional animal models in biomedical research. Three-dimensional bioprinting enables the creation of complex, patient-specific tissue models that mimic human physiology more accurately than animal models. These 3D bioprinted tissues, when integrated with microfluidic systems, can replicate the dynamic environment of the human body, allowing for the development of multi-organ models. This integration facilitates more precise drug screening and personalized therapy development by simulating interactions between different organ systems. Such innovations not only improve predictive accuracy but also address ethical concerns associated with animal testing, aligning with the three Rs principle. Future directions include enhancing bioprinting resolution, developing advanced bioinks, and incorporating AI for optimized system design. These technologies hold the potential to revolutionize drug development, regenerative medicine, and disease modeling, leading to more effective, personalized, and humane treatments.

## 1. Introduction

The field of bioengineering has made significant strides in recent years, particularly with the advent of 3D bioprinting and microfluidic lab-on-chip systems. However, despite these advancements, there are still considerable challenges in the realm of predictive modeling for drug screening and therapy development. Traditional animal models have long been the cornerstone of biomedical research, but their predictive accuracy is limited. Physiological differences between animals and humans can lead to discrepancies in drug efficacy and toxicity results. For instance, a drug that shows promising results in mice may fail in human trials due to differences in metabolism, immune response, and other biological factors [1,2]. This gap highlights the need for more accurate and human-relevant models. Three-dimensional bioprinting offers a groundbreaking solution to this problem. By using human cells, including patient-specific cells, researchers can create detailed and personalized tissue models [3]. Bioprinted structures can mimic the complex architecture and microenvironment of human tissues, providing more accurate responses to drug treatments [4]. This approach allows for personalized medicine, where therapies can be tailored to the individual patient’s cellular makeup, leading to more effective and targeted treatments [5]. Despite these advantages, most current 3D bioprinted models are limited to single organs [6,7]. Human physiology, however, is characterized by intricate interconnections between multiple organ systems. For instance, the liver’s role in drug metabolism affects the efficacy and toxicity of treatments on the heart, kidneys, and other organs [8]. Therefore, a comprehensive understanding of drug effects requires multi-organ models that can simulate these interactions. The integration of 3D bioprinting with microfluidic lab-on-chip systems addresses this limitation. Microfluidic devices can replicate the dynamic environment of the human body, including fluid flow, nutrient supply, and waste removal [9]. By combining these technologies, researchers can develop multi-organ systems that better reflect the complexity of human physiology, leading to more accurate drug screening and therapy development [10]. Thus, while traditional animal models have provided valuable insights, their limitations underscore the need for advanced bioengineering approaches. Three-dimensional bioprinting, especially when integrated with microfluidic systems, offers a promising path forward, enabling more detailed, personalized, and interconnected models that hold the potential to revolutionize biomedical research and clinical applications [11].

## 2. The Promise of 3D Bioprinting

3D bioprinting is a cutting-edge technology that constructs tissues and organs layer by layer using living cells. This method allows for the precise creation of complex biological structures that closely mimic natural tissues. Current applications range from skin grafts and cartilage repair to the development of more complex organs like bone, liver, and heart [12,13,14,15,16]. Compared to traditional methods, 3D bioprinting offers enhanced precision, customization, and the potential for patient-specific treatments. The technology behind 3D bioprinting has rapidly evolved, with several key techniques emerging as the most prominent. Extrusion-based bioprinting involves the use of a syringe or nozzle to deposit bioink layer by layer and is widely used due to its ability to print complex structures with high cell density, making it particularly suitable for creating large tissue constructs [4]. Inkjet bioprinting, similar to traditional inkjet printing, uses a printhead to deposit droplets of bioink onto a substrate. It is known for its high resolution and speed, making it ideal for creating precise, small-scale structures [4]. Laser-assisted bioprinting utilizes a laser to transfer bioink from a donor substrate to a collector substrate, offering high precision and the capability to print a wide variety of cell types and biomaterials, making it suitable for complex tissue engineering applications [17]. The materials used in 3D bioprinting, commonly referred to as bioinks, are crucial to the success of the printed structures. Bioinks must be biocompatible, support cell viability, and have appropriate mechanical properties. Hydrogels, such as alginate, gelatin, and hyaluronic acid, are widely used due to their high-water content and biocompatibility, providing a supportive environment for cell growth and being easily printable [18]. Decellularized extracellular matrix (dECM) bioinks are derived from natural tissues and organs that have been processed to remove cellular components. They retain the native biochemical cues of the original tissue, promoting cell attachment, proliferation, and differentiation [19]. Synthetic polymers like polyethylene glycol (PEG) and polycaprolactone (PCL) or nanomaterials like graphene or MXene are used for their tunable mechanical properties and ease of fabrication [20,21,22]. These polymers can be engineered to degrade at controlled rates, making them ideal for creating scaffolds that support tissue regeneration [23]. By using these advanced techniques and materials, researchers can create bioprinted structures that closely resemble native tissues. These structures provide more accurate responses to drug treatments and allow for personalized medicine, where therapies can be tailored to the individual patient’s cellular makeup, leading to more effective and targeted treatments.

## 3. Main Limitations of 3D Bioprinting Technology

Three-dimensional bioprinting, despite its potential for creating complex biological structures, faces several limitations. Extrusion-based bioprinting, suitable for large tissue constructs due to its ability to print high cell density structures, often suffers from lower resolution compared to other methods and can cause significant shear stress on cells, potentially affecting cell viability and function. The high viscosity of bioinks needed for extrusion can also limit the complexity of structures that can be printed. Inkjet bioprinting, known for its high resolution and speed, is limited by the types of bioinks it can use, requiring low-viscosity materials, which can restrict the range of materials and cell types that can be printed. Additionally, the droplet-based deposition method can lead to issues like clogging of nozzles and inconsistency in droplet size, affecting print quality and reproducibility. Laser-assisted bioprinting offers high precision and the ability to print a wide variety of cell types and biomaterials, but it is complex and expensive. The high-energy laser can also damage sensitive cells, reducing their viability. The materials used in 3D bioprinting, such as bioinks, must balance biocompatibility, cell viability, and appropriate mechanical properties, which can be challenging. Hydrogels provide a supportive environment for cell growth but may lack the mechanical strength required for certain applications, limiting their use in load-bearing tissues. Decellularized extracellular matrix (dECM) bioinks retain native biochemical cues but can vary significantly in composition and performance due to the variability in source tissues. Synthetic polymers and nanomaterials, like polyethylene glycol (PEG) and graphene, offer tunable mechanical properties and ease of fabrication but may require extensive optimization to support cell growth and function effectively. The degradation rates of these materials must also be carefully controlled to match the rate of tissue regeneration.

## 4. Microfluidic Lab-on-Chip Systems

Microfluidic lab-on-chip devices are miniaturized systems designed to simulate physiological conditions, enabling the study of biological processes in a highly controlled environment. These chips can replicate the dynamic nature of human tissues and organs by precisely controlling fluid flow, temperature, and chemical gradients on a microscale [24,25]. The core principle of microfluidic technology involves the manipulation of small volumes of fluids within networks of channels, often only tens to hundreds of micrometers in size. Microfluidic chips are typically fabricated using materials like polydimethylsiloxane (PDMS), glass, and thermoplastics [26]. PDMS is favored due to its biocompatibility, optical transparency, and flexibility, which make it ideal for creating complex channel structures [27]. Glass chips offer excellent chemical resistance and optical properties, while thermoplastics like polycarbonate provide durability and ease of mass production [28]. The fabrication processes for these chips include soft lithography for PDMS, photolithography for glass, and injection molding or hot embossing for thermoplastics [29]. One of the primary advantages of microfluidic lab-on-chip systems is the ability to control flows, volumes, and shear stress with high precision. This control allows for the accurate simulation of the microenvironment found in human tissues, which is crucial for studying cellular responses and drug effects [30]. Additionally, microfluidic chips can create gradients of chemicals or oxygen, mimicking the natural conditions cells experience in vivo [31]. The transparency of materials like PDMS and glass allows for real-time observation of biological processes under a microscope. This feature is particularly beneficial for fluorescence microscopy, enabling researchers to monitor cellular behavior, track drug diffusion, and observe interactions at a cellular level. Furthermore, integrating electrodes within the microfluidic channels can facilitate the monitoring of biochemical changes, such as pH and oxygen levels, providing valuable insights into cellular metabolism and responses to treatments [32]. The combination of these features makes microfluidic lab-on-chip devices powerful tools in biomedical research. They are currently used in drug testing, disease modeling, and personalized medicine, offering high-throughput and cost-effective solutions [33,34]. By providing a controlled and replicable environment, these chips enable more accurate predictions of how drugs will perform in human tissues, reducing the reliance on animal models and accelerating the development of new therapies.

## 5. Main Limitations of Microfluidic Lab-on-Chip Systems

Microfluidic lab-on-chip devices, while powerful tools in biomedical research, present several limitations. The fabrication processes for these chips, including soft lithography for PDMS, photolithography for glass, and injection molding or hot embossing for thermoplastics, can be complex, time-consuming, and require specialized equipment. PDMS, although biocompatible and flexible, can absorb small hydrophobic molecules, potentially affecting experimental outcomes and the reproducibility of results. Glass chips offer excellent chemical resistance and optical properties but are fragile and can be difficult to handle and integrate with other systems. Thermoplastics like polycarbonate provide durability and ease of mass production, but they may lack the optical clarity and chemical resistance needed for certain applications. Achieving precise control over fluid flow, shear stress, and chemical gradients in microfluidic systems is crucial for accurately simulating the microenvironment of human tissues, but this can be technically challenging. Maintaining these conditions over extended periods and ensuring uniform distribution of fluids and nutrients can be difficult. Additionally, while the transparency of materials like PDMS and glass allows for real-time observation of biological processes, integrating sensors and electrodes within the microfluidic channels to monitor bio-chemical changes adds complexity to the chip design and fabrication process. This integration is essential for monitoring parameters like pH, oxygen levels, and cellular metabolism, which provide valuable insights into cell behavior and responses to treatments. Despite these challenges, the high-throughput and cost-effective solutions provided by microfluidic lab-on-chip devices make them valuable for drug testing, disease modeling, and personalized medicine, although further advancements in fabrication techniques, materials, and integration strategies are needed to fully realize their potential.

## 6. Integration of 3D Bioprinting and Microfluidics

The integration of 3D bioprinting and microfluidic lab-on-chip technologies represents a significant leap forward in creating advanced multi-organ systems. This combination leverages the strengths of both technologies to produce complex, physiologically relevant models that can simulate the interactions between different tissues and organs. By integrating these systems, researchers can develop more accurate and dynamic models for studying human biology and disease.

### 6.1. Advantages of Integration

Integrating 3D bioprinting with microfluidics offers several advantages. Firstly, it enhances the physiological relevance of in vitro models by mimicking the architecture and microenvironment of human tissues more accurately than traditional methods. This improved mimicry leads to better predictive models for drug testing and disease research. Secondly, the dynamic nature of microfluidic systems allows for the precise control of fluid flow, shear stress, and chemical gradients, which are crucial for maintaining cell viability and function in bioprinted tissues. Thirdly, the integration facilitates the creation of multi-organ systems that can simulate complex inter-organ interactions, providing a more comprehensive understanding of systemic responses to drugs and disease processes.

### 6.2. Principles and Underlying Mechanisms

Combining 3D bioprinting with microfluidics involves several key steps. Initially, 3D bioprinting is used to fabricate tissue constructs with precise architecture and cellular composition. These bioprinted tissues can include multiple cell types arranged in specific patterns to mimic the organization of native tissues. Various biomaterials are utilized, such as hydrogels for soft tissues or synthetic polymers for more rigid structures. For example, bioprinting can create a liver tissue with hepatocytes and endothelial cells within a hydrogel matrix while simultaneously printing cardiac tissue composed of cardiomyocytes and fibroblasts [35,36]. Once the tissues are printed, they are integrated onto a microfluidic chip that provides the necessary fluidic environment to sustain and interconnect the tissues. The microfluidic system controls the flow of culture media, delivering nutrients and oxygen while removing waste products, thus mimicking the circulatory system. The ability to control shear stress, fluid flow, and chemical gradients within the microchannels is crucial for maintaining tissue viability and function. Creating multi-organ systems involves connecting several bioprinted tissues on a single microfluidic platform, allowing them to interact and communicate with each other.

### 6.3. Examples of Applications

One notable example is the development of a liver–heart–kidney chip, where each organ is bioprinted and then connected via microfluidic channels. This setup allows researchers to study the systemic effects of drugs, including metabolism by the liver, cardiovascular responses, and renal clearance, providing a more comprehensive understanding of drug interactions and toxicity. Another significant application is in cancer research, particularly in studying primary tumors and secondary metastases. By bioprinting a primary tumor model and connecting it to a secondary organ model via a microfluidic system, researchers can observe the process of metastasis in real-time. This setup enables the study of how cancer cells migrate from the primary site, invade secondary tissues, and how the microenvironment of the secondary site influences tumor growth and progression. The integration of 3D bioprinting and microfluidics also facilitates the creation of complex biological barriers, such as the intestinal barrier [37]. The intestinal barrier is characterized by its intricate villi structures, which can be challenging to replicate. However, 3D bioprinting can accurately recreate these structures with detailed morphology. When combined with microfluidic systems, these bioprinted intestinal models can simulate the dynamic flow of digestive fluids and nutrients, as well as the microbial interactions within the gut. This setup is particularly valuable for studying the gut–brain axis, where researchers can observe how changes in the gut environment affect neurological functions and vice versa.

### 6.4. Mechanistic Insights

The underlying mechanisms of this integration involve several critical factors. The precise control of cell placement and the architecture of the bioprinted tissues ensure that the structural and functional properties of native tissues are closely mimicked [38]. Microfluidic systems provide a controlled environment that can simulate physiological conditions such as fluid dynamics, mechanical forces, and chemical gradients. This combination allows for real-time monitoring and adjustment of the microenvironment, ensuring optimal conditions for cell growth and function. Additionally, the ability to create complex tissue interfaces and multi-organ systems enables the study of inter-organ communication and systemic biological responses, providing deeper insights into human physiology and disease mechanisms [39].

## 7. Reducing Animal Models in Research

The scientific limitations and ethical considerations associated with animal models in biomedical research have prompted a significant push towards alternative methods.

### 7.1. Scientific Limitations

While animal models have traditionally been instrumental in understanding human biology and disease, their predictive accuracy for human outcomes is often limited. Physiological and genetic differences between animals and humans can result in discrepancies that undermine the translational value of animal studies. One of the primary issues with animal models is the high rate of failure in clinical trials despite promising preclinical results. For example, studies have shown that around 90% of drugs that pass animal testing fail in human trials due to ineffectiveness or unexpected toxicity [40]. A well-known case is that of Alzheimer’s disease research, where numerous compounds have shown efficacy in animal models but failed to produce beneficial effects in human clinical trials [41]. Similarly, cancer treatments that appear promising in rodent models often do not translate into successful human therapies due to differences in tumor biology and the immune response [42]. Several physiological and pathological discrepancies contribute to these failures. For instance, the metabolism of drugs can vary significantly between species. Drugs metabolized effectively by rodents may be processed differently by human liver enzymes, leading to variations in efficacy and toxicity. The immune system also presents notable differences; animals often do not replicate the complexity of human immune responses, affecting the outcomes of immunotherapies and vaccines. Additionally, the genetic diversity of human populations contrasts with the homogeneous genetic backgrounds of laboratory animals, impacting the generalizability of animal research findings.

### 7.2. Ethical Considerations

The ethical framework guiding the reduction of animal use in research is encapsulated by the three Rs principle: Replacement, Reduction, and Refinement. This principle encourages scientists to seek alternatives to animal models, minimize the number of animals used, and refine experimental procedures to minimize suffering. Regulatory bodies and research institutions globally advocate for these practices, emphasizing the moral imperative to reduce animal suffering and the scientific necessity for more human-relevant models [43]. In response to these ethical and scientific challenges, multi-organ systems created through the integration of 3D bioprinting and microfluidic technologies offer promising alternatives [38]. Furthermore, recent policies and bioethical guidelines increasingly recommend the minimization of animal use in research. The European Union’s Directive 2010/63/EU on the protection of animals used for scientific purposes is one such example, mandating the consideration of alternative methods before resorting to animal testing. In the United States, the National Institutes of Health (NIH) has implemented the “Alternatives to Animal Testing” initiative, promoting the development and use of non-animal methods in research [44,45]. The push to reduce animal models in research is driven by both ethical imperatives and the need for more reliable human-relevant data. By adopting these innovative approaches, the scientific community can improve the translational success of research findings and reduce the ethical burden associated with animal testing.

## 8. Challenges and Future Directions

### 8.1. Technical and Practical Challenges in the Integration of Technologies

Integrating 3D bioprinting with microfluidic lab-on-chip systems presents several technical and practical challenges. One of the primary technical hurdles is maintaining cell viability and function during and after the bioprinting process. The mechanical forces and environmental conditions involved in bioprinting can stress cells, potentially affecting their viability and behavior. Ensuring that cells maintain their intended functions within the bioprinted structures is crucial for the success of these models [46]. Another significant challenge is achieving precise control over the microenvironment within the microfluidic systems. This includes regulating fluid flow, shear stress, and chemical gradients to accurately mimic physiological conditions. The complexity of replicating the dynamic interactions between multiple tissues and organs also poses difficulties. For instance, ensuring consistent communication and nutrient exchange between interconnected tissues requires sophisticated design and engineering of both the bioprinted structures and the microfluidic channels [47]. Scalability and reproducibility are additional concerns. Producing consistent and reliable multi-organ systems at a scale suitable for high-throughput screening is challenging. Variations in the fabrication process can lead to differences in the properties and behavior of the bioprinted tissues and microfluidic systems, potentially impacting experimental outcomes [48,49,50,51].

### 8.2. Potential Future Developments and Innovations

Future developments in this field are likely to focus on improving the resolution and functionality of 3D bioprinting and microfluidic technologies. Advances in bioink formulations, including the development of more sophisticated and tissue-specific biomaterials, will enhance the fidelity and functionality of bioprinted tissues. Innovations in bioprinting techniques, such as the use of multi-material and multi-cell-type printing, will enable the creation of more complex and realistic tissue models [52]. On the microfluidic front, future innovations may involve the integration of more advanced sensing and monitoring capabilities. For example, incorporating real-time monitoring of multi biochemical markers could provide deeper insights into the physiological states of the bioprinted tissues. Additionally, advancements in automation and robotics could streamline the fabrication and maintenance of these systems, improving scalability and reproducibility [53].

Integrating bioprinting technology at the microscale with microfluidic lab-on-chip systems requires significant advancements. Enhancing bioprinting resolution is crucial; this can be achieved by utilizing high-precision inkjet and laser-assisted bioprinting techniques capable of sub-micron level deposition. Specialized bioinks must also be developed with appropriate viscosity and cell compatibility for precise microscale printing, incorporating materials such as hydrogels and decellularized extracellular matrices to support the microenvironment. Additionally, developing modular microfluidic platforms using photolithography and soft lithography techniques will ensure compatibility and functionality. Achieving these advancements will significantly enhance the creation of detailed and functional micro-tissues. This integration holds the potential for more accurate disease models, improved drug screening, and advances in personalized medicine. Focusing research on high-resolution bioprinting, innovative bioink formulations, and precise microfluidic control will be essential to realize these possibilities fully.

Emerging technologies such as artificial intelligence (AI) and machine learning could play a crucial role in optimizing the design and operation of multi-organ systems. AI algorithms can be used to model and predict the behavior of complex biological systems, guiding the design of more effective bioprinted tissues and microfluidic setups. Machine learning can also assist in analyzing the vast amounts of data generated by these systems, identifying patterns and insights that may not be immediately apparent [54,55,56,57,58].

### 8.3. Long-Term Impact on Biomedical Research and Clinical Applications

The long-term impact of integrating 3D bioprinting and microfluidic technologies in biomedical research and clinical applications is profound. These systems have the potential to revolutionize drug development by providing more accurate and human-relevant models for preclinical testing. This could significantly reduce the time and cost associated with bringing new drugs to market, as well as increase the success rate of clinical trials by providing better predictive data on drug efficacy and safety [12,37,59,60]. In regenerative medicine, these technologies could lead to breakthroughs in the development of bioengineered tissues and organs for transplantation. The ability to create patient-specific tissues could reduce the risk of immune rejection and improve the outcomes of transplantation procedures. Furthermore, multi-organ systems could be used to study complex diseases that involve multiple organ systems, such as diabetes, cancer, and neurodegenerative disorders, leading to new therapeutic approaches and personalized medicine strategies [61]. Overall, the integration of 3D bioprinting and microfluidic technologies represents a transformative advancement in biomedical research and clinical applications. By overcoming the current technical and practical challenges and embracing future innovations, these systems hold the promise of significantly advancing our understanding of human biology and improving healthcare outcomes.

## 9. Conclusions

The integration of 3D bioprinting and microfluidic technologies is set to revolutionize bioengineering, offering precise replication of human physiology for more accurate drug testing, personalized medicine, and innovative therapies. This advancement addresses ethical concerns and scientific limitations of animal models, aligning with the 3Rs principles and enhancing the predictive power of preclinical studies. Looking forward, the future of bioengineering will likely involve further integration of artificial intelligence and machine learning to optimize the design and functionality of multi-organ systems. These technologies will enable sophisticated modeling of complex diseases, providing novel insights and treatment strategies. The development of new biomaterials will enhance the fidelity and functionality of bioprinted tissues, while increasingly complex systems will better replicate biological complexity and specific pathologies. As these technologies evolve, they will gradually replicate the intricate interactions of human biology more faithfully. This promises a future where medical treatments are more effective, personalized, and humane, marking a significant leap forward in biomedical research and clinical applications. The bioengineering field is on the cusp of a new era, poised to make substantial contributions to human health and well-being.
